# Numerical simulation of mitochondrial systems for ATP generation and membrane transport

**DOI:** 10.1038/s41598-026-61463-0

**Published:** 2026-07-20

**Authors:** Takashi Sato, Tsuyoshi Osawa

**Affiliations:** 1 Research & Development Center, Zeon Corporation, 210- 9507 Kanagawa, Japan; 2 Division of Integrative Nutiriomics and Oncology, Research Center for Advanced Science and Technology, The University of Tokyo, Tokyo, 153-8904 Japan; 3 Department of Chemistry and Biotechnology, Graduate School of Engineering, The University of Tokyo, Tokyo, 113-0033 Japan; 4 Department of Biological Sciences, Graduate School of Science, The University of Tokyo, Tokyo, 113-0033 Japan

**Keywords:** Natural number simulation, Membrane transport proteins, Metabolic modeling, stoichiometric equations, Glycolysis, Tricarboxylic acid cycle, Mitochondrial ATP synthesis, Biochemistry, Biophysics, Computational biology and bioinformatics, Mathematics and computing, Systems biology

## Abstract

Simulating intracellular biochemical reactions remains a significant challenge in mathematical modeling because of the complex interactions among diverse molecular species. The natural number simulation (NNS) framework offers a dynamic approach to simulating these reactions using a novel algorithm based on reaction equations. In this study, we developed a computational cell model incorporating mitochondria to examine key metabolic processes, including glucose uptake, glycolysis, the tricarboxylic acid cycle, and ATP synthesis via the electron transport chain. Substrate transport mediated by membrane proteins, such as pyruvate and nicotinamide adenine dinucleotide transporters, and the electron transport chain, was replicated using simplified reaction equations. The simulation results showed that, with appropriately chosen rate constants, the ATP production rate reached approximately **155 molecules s**^**− 1**^ **per ATP synthase**. Sensitivity analysis indicated that the number of mitochondrial phosphate transporters and the rate of phosphate transport into mitochondria strongly influence ATP production. The model also showed that intermittent glucose supply has a minimal impact on ATP production and that the framework is capable of incorporating the effects of deuterium-containing water on ATP synthesis. This framework provides a foundation for future efforts in simulating more detailed metabolic pathways and integrating experimental data.

**Supplementary Information:**

The online version contains supplementary material available at https://doi.org/10.1038/s41598-026-61463-0.

## Introduction

Intracellular biochemical reactions are highly complex and involve numerous molecular species, making their mathematical modeling particularly challenging. Advances in omics technologies and single-cell analyses have dramatically increased both the scale and complexity of biological data^1–3^. Machine learning and deep learning models are among the emerging approaches that have gained considerable attention in advancing current understanding of intracellular processes^4–6^. These developments have heightened the demand for computational models that can accurately simulate biochemical systems^7,8^.

Metabolic flux analysis has long been used to quantify cellular metabolic states. Techniques such as 13 C tracer analysis and flux balance analysis are powerful tools for predicting and quantifying metabolite flow through central pathways, including glycolysis, the tricarboxylic acid (TCA) cycle, and the electron transport chain^9–13^.

Traditionally, these dynamics are described using ordinary differential Eqs^14–16^. However, formulating reaction rate equations is highly complex, presenting challenges in scalability across cell types and in simplifying model structures^8,17^.

Several stochastic simulation approaches have been developed to model biochemical reaction networks, including the Gillespie stochastic simulation algorithm^14^, stochastic differential equation–based methods^18^, and stochastic Petri nets^19^. These approaches provide rigorous frameworks for simulating stochastic biochemical dynamics. However, in highly structured systems involving multiple compartments and transport processes, constructing explicit stochastic rate equations or master equations can become complex. The natural number simulation (NNS) framework adopts a different approach by directly evaluating reaction events using binomial probabilities and hierarchical domain structures^20^. This formulation enables complex compartmental systems to be implemented directly from reaction equations without explicitly deriving stochastic rate equations.

The primary advantage of NNS is its ability to explicitly model small populations of molecular species, such as genes, by directly manipulating molecular counts. Moreover, NNS can incorporate enzymes and membrane proteins into reaction equations to yield a more intuitive representation.

One potential advantage of the NNS framework is that reaction events are computed directly from discrete molecular numbers rather than continuous concentrations. In cellular systems, many molecular species, including enzymes and membrane transporters, exist in relatively small copy numbers, and their stochastic fluctuations can significantly affect metabolic dynamics. By explicitly tracking molecular counts and reaction stoichiometry, the NNS framework can naturally represent these discrete effects without requiring additional stochastic terms or approximations. This feature may enable the model to reproduce biologically realistic behaviors, particularly in compartmentalized systems where transport processes and enzyme abundances play a crucial role in determining metabolic fluxes.

In this study, we used the NNS framework to examine the influence of membrane proteins on intracellular dynamics through substrate transport. Membrane proteins frequently mediate exchange between compartments, such as the cytosol and mitochondria, and often represent rate-limiting steps in metabolic regulation; thus, accurately modeling their function is critical^21–23^. To demonstrate this, we constructed a virtual eukaryotic cell containing a single mitochondrion. We simulated key processes, including glucose uptake, glycolysis, the TCA cycle, and ATP synthesis, via the electron transport chain.

The Python program and input files used in this study are publicly available under the MIT License at the following GitHub repository: https://github.com/taka-b/binomial/tree/f3d59ac (branch: binomial_v018_02).

## Methods

### Introduction to the NNS framework

This study considers a hierarchical system consisting of a cell with a mitochondrion contained within it. In the NNS framework, such cellular compartments, including the cytoplasm and mitochondria, are represented as sets that define hierarchical domains. Molecular species are labeled according to their location inside or outside a compartment.　Simulations are performed using plain-text input files containing the basic settings required for the calculation, including simulation time, molecular elements, reaction definitions, and plotting instructions.

Molecular species are labeled according to their location inside or outside a compartment. For instance, extracellular glucose is denoted simply as glucose, whereas intracellular glucose is expressed as glucose:=. The symbol “:=” indicates intracellular localization. This notation is also used with membrane-associated proteins, such as glucose transporters.

The symbol “::” is used to indicate molecules localized within mitochondria. Reaction equations also adopt the same compartment-specific notations for consistency. This scheme allows the same molecular species to participate in different reactions depending on the compartment in which they are located.

**Fig. 1:**
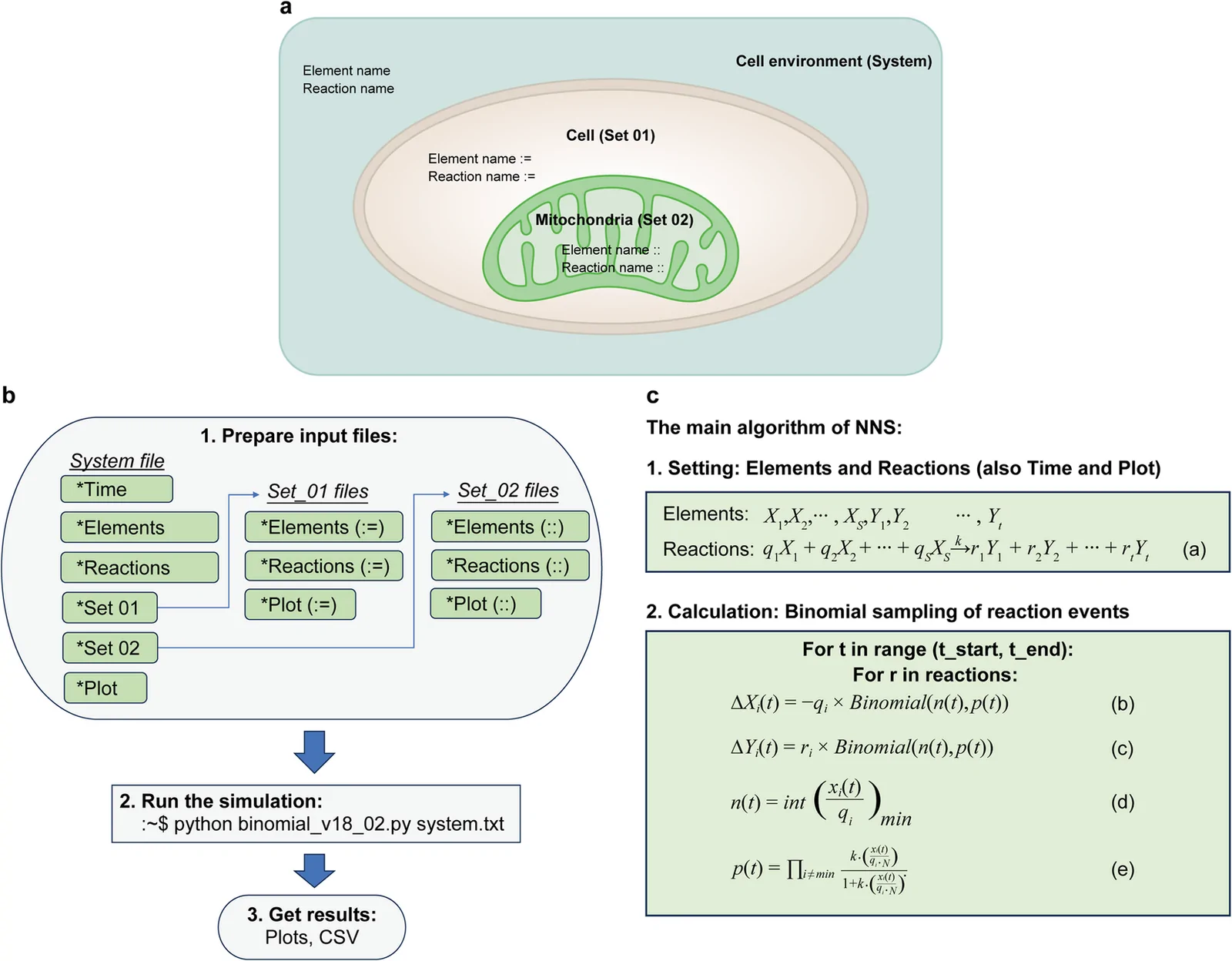
Concept and the natural number simulation (NNS) framework. (**A**) System model at the first and second hierarchical levels of the natural number simulation framework. The figure shows a single cell defined at the first level (Set01) and a mitochondrion within that cell at the second level (Set02). Elements and reactions are independently specified in the extracellular region (cell environment), cell, and mitochondrion. (**B**) Workflow of the simulation framework. Input files describing elements, reactions, parameter sets, and plotting conditions are prepared and processed by the Python simulation program to generate output data, such as plots and CSV files. (**C**) Main algorithm of the NNS framework.(**a**) Definition of elements and reactions.

Figure 1A illustrates the hierarchical structure of the NNS framework. The system domain contains a single cell defined at the first hierarchy level (Set01), within which a mitochondrion is defined at the second hierarchy level (Set02). Elements and reactions are independently specified in each domain (extracellular space, cell, and mitochondrion). The intercompartmental exchanges of molecular elements are defined across hierarchical levels.

Figure 1B shows the types of input files required for the simulation and the overall computational workflow. In this study, three files are required because the model consists of the extracellular environment, the cell, and the mitochondrion. However, the simulation is executed in the same manner as in previous implementations by running the main system file.

Figure 1C presents a simplified overview of the NNS algorithm. The fundamental algorithm is identical to that described in the original publication^20^. Because the simulation consists of three hierarchically structured files, the elements defined in these files are first merged into a unified list, and all reactions are collected into a single set of reaction equations as represented in Eq. (a) of Fig. 1C. At each time step, the quantities shown in Eqs. (b)–(c) of Fig. 1C are calculated for all defined reactions, yielding the changes in molecular counts, ΔX_i_(t) and ΔY_i_(t). In the implementation, **Binomial(n**,** p)** represents a random value sampled from a binomial distribution. As shown in Eqs. (d)–(e), the number of trials *n* depends on the current molecular number X_i_(t) and the reaction order q_i_, whereas the probability *p* depends on X_i_(t), q_i_, the rate constant *k*, and the normalization constant *N*. When multiple reactions compete for the same molecular species, reactions are evaluated sequentially within each simulation step. To avoid systematic bias arising from a fixed update order, the list of reactions is randomly shuffled at every time step before evaluation. As an optional setting, a fixed reaction order can also be specified when required for certain analyses.

**Fig. 2:**
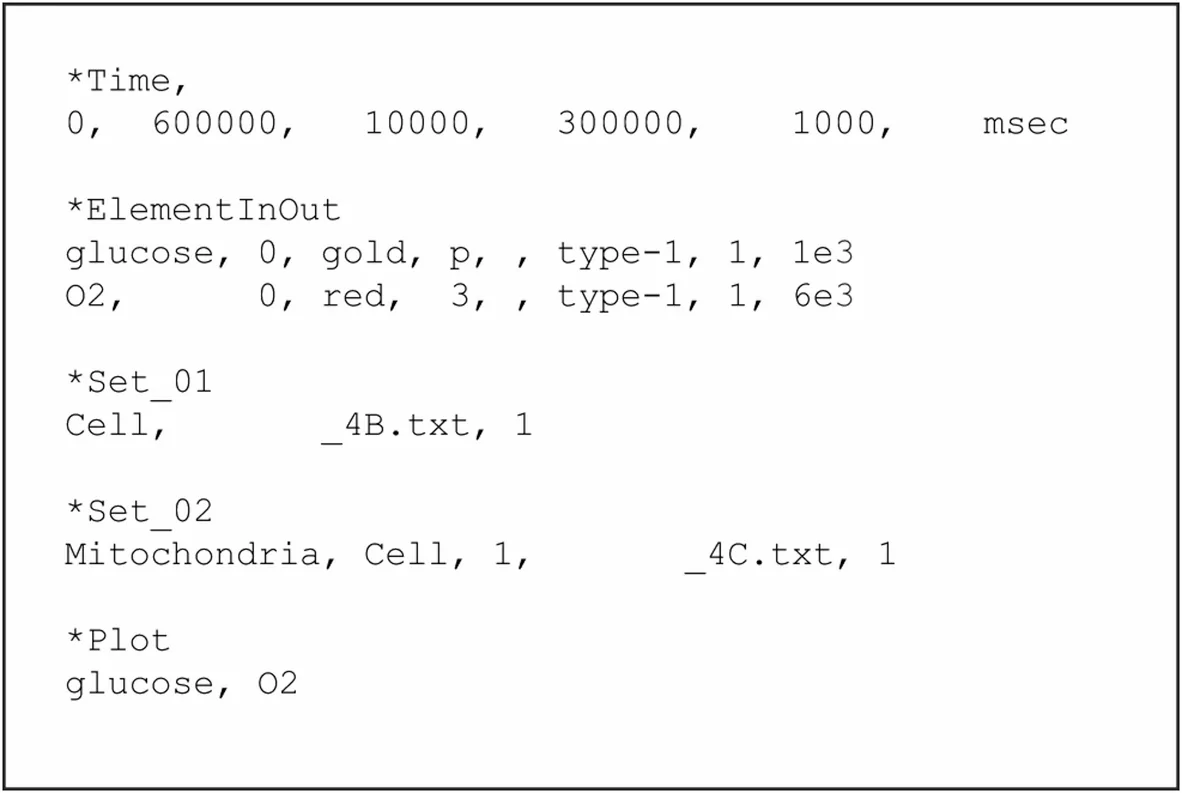
Input file for a two-layer system. The *Time* statement specifies the calculation steps from 0 to 600,000 with a time interval of 1 ms. Glucose and O₂ are supplied to the environment at rates of 1 × 10^3^ and 6 × 10^3^ molecules per time step, respectively, as defined in the *ElementInOut* section. The *Set_01* and *Set_02* files are also referenced in this file. The *Plot* statement generates a graph of glucose and O_2_ dynamic.

Figure 2 presents an example of a system input file used in this study. In the NNS framework, most quantities are defined as dimensionless values. Therefore, an explicit time unit must be specified to relate the simulation time to physical time. In the *Time section of the input file shown in Fig. 2, the unit “msec” is specified, meaning that one simulation step corresponds to 1 ms of physical time. Because the number of steps is defined from 0 to 600,000, the simulation corresponds to a duration of 600 s. The input file follows the same hierarchical format applied in the simulations, where the cell corresponds to the Set01 domain (*Set_01*), and the mitochondrion corresponds to the Set02 domain (*Set_02*). Under the *ElementInOut* section, glucose and oxygen are depicted as continuously entering the system. The complete input file is provided in Supplementary Fig. 1.

### Membrane transport reactions and the definition of the first hierarchy (Set01)

The intracellular reactions included in the first hierarchy are summarized in Table 1, with the major ones described below. In the NNS framework, reactions are expressed using stoichiometry-like equations (100:=), such as the following:

**Table 1:** Cellular reactions and their names, reactants, products, and rate constants.

Reaction name	Reactants	Rate constant	Products
10:=	H_2_O:=	1e − 6	H+:= + OH−:=
20:=	H+:= + OH−:=	1e4	H_2_O:=
100:=	glucose_transport:= + 10 glucose	1e9	glucose_transport:= + 10 glucose:=
200:=	O_2_	0.1	O_2_:=
300:=	glucose:= + 2 ATP:= + 2 ADP:= + 2 Pi:= + 2 NAD+:=	1e4	2 Pyruvate−:= + 4 ATP:= + 2 NADH:= + 4 H+:= + 2 H_2_O:=
400:=	Pyruvate−:= + NADH:= + H+:=	1e3	Lactic-:= + NAD+:=
500:=	ATP:= + H_2_O:=	1e − 4	ADP:= + Pi:=

All rate constants are dimensionless and reported as numerical values. H_2_O, water; H+, proton; OH−, hydroxide ion; glucose_transport, glucose transporter; ATP, adenosine triphosphate; ADP, adenosine diphosphate; Pi, inorganic phosphate; NAD+, nicotinamide adenine dinucleotide (oxidized form); NADH, nicotinamide adenine dinucleotide (reduced form).

1 $$glucose{\text{ }} + {\text{ }}glucose\_transport: = {\text{ }} \to {\text{ }}glucose: = {\text{ }} + {\text{ }}glucose\_transport: =$$

Here, glucose_transport:= functions as a catalytic molecule that mediates glucose transport from the extracellular environment into the cytoplasm^23,24^. Its appearance on both sides of the equation reflects its catalytic role. The algorithm allows the equation to be written in this format to accurately calculate the number of molecules.

Oxygen diffusion (200:=) represents passive transport across the membrane, occurring without the involvement of specific transport proteins. It is depicted by changing the notation from extracellular to cytoplasmic:

2 $${\mathrm{O}}_{{\mathrm{2}}} \to {\text{ O}}_{{\mathrm{2}}} : =$$

Although only the unidirectional reaction is shown here, the reverse reaction can also be defined if necessary^25–27^. The principal glycolytic reaction is represented as follows (300:=):

$$glucose: = {\text{ }} + {\text{ }}2{\text{ }}ATP: = {\text{ }} + {\text{ }}2{\text{ }}ADP: = {\text{ }} + {\text{ }}2{\text{ }}Pi: = {\text{ }} + {\text{ }}2{\text{ }}NAD + : = {\text{ }} \to$$

3 $$2{\text{ }}pyruvate - : = {\text{ }} + {\text{ }}4{\text{ }}ATP: = {\text{ }} + {\text{ }}2{\text{ }}NADH: = {\text{ }} + {\text{ }}4{\text{ }}H + : =$$

In the NNS framework, reaction orders must be natural numbers. Since glycolysis requires ATP for progression, ATP is listed among the substrates. Consequently, two molecules of ATP are produced per glucose molecule. Although enzymes are not explicitly indicated in this representation, they can be specified if needed^28,29^.

Additional reactions in this hierarchy include water ionization (10:=), water production (20:=), lactate formation (400:=), and ATP hydrolysis (500:=) (Table 1). The Set01 file shown in Fig. 2 can be generated as an input file by making minor modifications to the equations in Table 1. The final input file is provided in Supplementary Fig. 2.

### Reactions in the mitochondria

The mitochondrial reactions examined in this study are summarized in Table 2, with the principal reactions described below. Pyruvate (*pyruvate−:=*), which is generated in the cytoplasm, is imported into the mitochondrion through the (*pyruvate_transporter*::). This process is represented as follows:

**Table 2:** Mitochondria reactions and their names, reactants, products, and associated arate constants.

Reaction name	Reactants	Rate constant	Products
10::	H_2_O::	1e − 6	H+:: + OH−::
20::	H+:: + OH−::	1e4	H_2_O::
100::	Pyruvate_transport:: + Pyruvate−:= + H+:=	1e5	Pyruvate_transport:: + Pyruvate−:: + H+::
200::	Pyruvate−:: + CoA-SH:: + NAD+::	1e3	Acetyl_CoA:: + NADH:: + H+:: + CO_2_::
210::	Acetyl_CoA:: + 3 NAD+:: + GDP:: + Pi:: + 3 H_2_O:: + FAD::	1e3	CoA-SH:: + 3 NADH:: + 3 H+:: + GTP:: + 2 CO_2_:: + FADH2::
220::	GTP:: + ADP::	1e1	GDP:: + ATP::
300::	2 NADH:: + 222 H+:: + O_2_:: + Commp1-3-4::	1e5	2 NAD+:: + 20 H+_inter:: + 2 H_2_O:: + Commp1-3-4::
310::	2 FADH2:: + 22 H+:: + O_2_:: + Commp2-3-4::	1e5	2 FAD:: + 12 H+_inter:: + 2 H_2_O:: + Commp2-3-4::
320::	12 H+_inter:: + 3 ADP:: + 3 Pi:: + ATP_synthase::	6e3	12 H+:: + 3 ATP:: + ATP_synthase::
400::	NADH:= + H+:= + NAD+:: + NADH_shuttle::	1e3	NADH:: +H+:: + NAD+:= + NADH_shuttle::
500::	Pi_transport:: + Pi:= + H+_inter::	1e2	Pi_transport:: + Pi:: +H+::
600::	ADP_ATP_transport:: + ATP:: + ADP:=	1e4	ADP_ATP_transport:: + ATP:= ADP::
700::	O_2_:=	1e1	O_2_::

All rate constants are dimensionless and expressed as numerical values.

4 $${\mathrm{pyruvate}} - : = {\text{ }} + {\text{ H}} + : = {\text{ }} + {\text{ pyruvate}}\_{\mathrm{transporter}}::{\text{ }} \to {\text{ pyruvate}} - ::{\text{ }} + {\text{ H}} + ::{\text{ }} + {\text{ pyruvate}}\_{\mathrm{transporter}}::$$

This equation describes the cotransport of pyruvate and protons from the cytoplasm (pyruvate−:= and H+:=) into the mitochondrial matrix (pyruvate−:: and H+::)^30–34^.

Although the TCA cycle is one of the central mitochondrial pathways, it is expressed here in a simplified, aggregated form (210::):

$$acetyl\_CoA::{\text{ }} + {\text{ }}3{\text{ }}NAD + ::{\text{ }} + {\text{ }}GDP::{\text{ }} + {\text{ }}Pi::{\text{ }} + {\text{ }}3{\text{ }}H_{2} O::{\text{ }} + {\text{ }}FAD::{\text{ }} \to$$

5 $$CoA - SH::{\text{ }} + {\text{ }}3{\text{ }}NADH::{\text{ }} + {\text{ }}3{\text{ }}H + ::{\text{ }} + {\text{ }}GTP::{\text{ }} + {\text{ }}2{\text{ }}CO_{2} ::{\text{ }} + {\text{ }}FADH2::$$

The definitions of these components are provided in the footnotes of Table 2^35–37^. A unique feature of the NNS framework is its ability to accommodate reactions of any complexity, as there are no restrictions on the number of reactants or products. Thus, even multistep processes can be expressed as a single reaction equation.

The translocation of H^+^ into the mitochondrial intermembrane space via the electron transport chain is expressed as:

$${\text{2 NADH}}::{\text{ }} + {\text{ 22 H}} + ::{\text{ }} + {\text{ O}}_{{\mathrm{2}}} ::{\text{ }} + {\text{ Commp1}} - {\mathrm{3}} - {\mathrm{4}}::{\text{ }} \to$$

6 $${\text{2 NAD}} + ::{\text{ }} + {\text{ 2}}0{\text{ H}} + \_{\mathrm{inter}}::{\text{ }} + {\text{ 2 H}}_{{\mathrm{2}}} {\mathrm{O}}::{\text{ }} + {\text{ Commp1}} - {\mathrm{3}} - {\mathrm{4}}::$$

Here, *Commp1-3-4*:: represents an aggregate model of NADH: ubiquinone oxidoreductase (Complex I), the cytochrome bc1 complex (Complex III), and cytochrome c oxidase (Complex IV) of the mitochondrial inner membrane. *H+_inter*:: denotes the protons translocated by these complexes from the mitochondrial matrix into the intermembrane space. Similar to Eq. (4), this reaction involves a large-scale proton flux, which defines the reaction rate per unit time, expressed in ms^− 1^^38–40^. Furthermore, the reaction catalyzed by ATP synthase is expressed as follows:

$$12{\text{ }}H + \_inter::{\text{ }} + {\text{ }}3{\text{ }}ADP::{\text{ }} + {\text{ }}3{\text{ }}Pi::{\text{ }} + {\text{ }}ATP\_synthase::{\text{ }} \to$$

7 $$12{\text{ }}H + ::{\text{ }} + {\text{ }}3{\text{ }}ATP::{\text{ }} + {\text{ }}ATP\_synthase::$$

This equation indicates that during each calculation step (1 ms in this study), a single ATP synthase molecule (*ATP_synthase*::) transfers 12 intermembrane protons (*H+_inter*::) back into the matrix, resulting in ATP production. Since one ATP molecule is synthesized from four protons, the reaction is stoichiometrically consistent^41–43^.

Other mitochondrial reactions are summarized in Table 2. In addition to Eq. (7), these mitochondrial reactions include water ionization (10::) and production (20::); conversion of pyruvate to acetyl-CoA (200::); the TCA cycle (210::); ATP production from GTP (220::); proton translocation mediated by FADH2 and the aggregate *Commp2-3-4*:: (defined as a combination of succinate dehydrogenase, the cytochrome b-c1 complex, and cytochrome c oxidase) (310::); the exchange between cytosolic NADH and mitochondrial NAD+ (400::)^44–46^; phosphate ion transport (500::); and ATP transport (600::). The specific input file is shown in Supplementary Fig. 3.

## Results

The computational results were obtained using the input files corresponding to Supplementary Figs. 1–3, along with results from slightly modified conditions. These three files were integrated during execution within the NNS framework, after which the calculations were performed.

In the unified file, the elements and reaction names defined in *Set_01 were modified to include the tag “Cell_1.” Here, “_1” indicates that a single cell was generated in Set01 based on the original file. For example, glucose:= becomes glucose:=Cell_1, and reaction 100:= becomes 100:=Cell_1. Similarly, the elements and reactions defined in *Set_02 were modified to include “:: Cell_1::Mitochondria_1.” For instance, pyruvate in the mitochondria, written initially as Pyruvate−::, is renamed Pyruvate−::Cell_1::Mitochondria_1 in the integrated file. Reaction names are modified in the same manner.

**Fig. 3:**
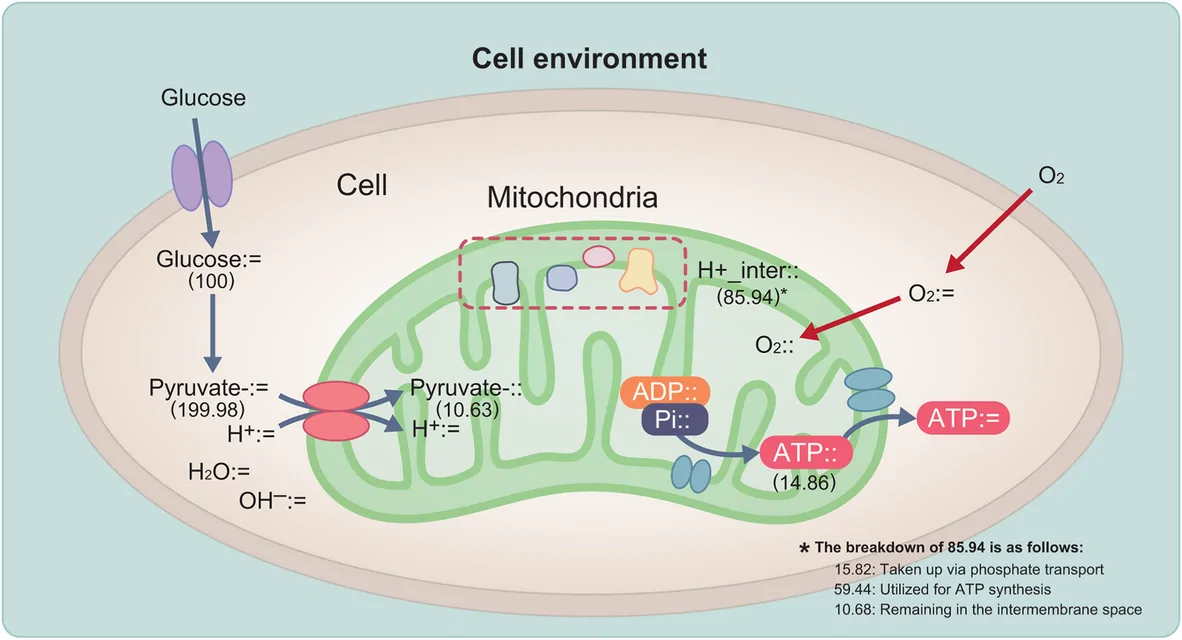
Symbolic representation of the major molecules in the model according to their cellular locations. Molecules outside the cell lack a suffix, as seen in glucose and O_2_. Molecules within the cytoplasm are indicated by the suffix “:=”. H+_inter:: represents protons in the mitochondrial intermembrane space. These protons are actively transported out of the mitochondrial matrix via the electron transport chain. The values displayed above each molecular species indicate the cumulative amounts produced and normalized to a total intracellular glucose uptake of 100.

As illustrated in Fig. 3, glucose and oxygen are initially supplied to the extracellular environment and subsequently transported into the cell. In the cytoplasm, glucose undergoes glycolysis, generating pyruvate as the main product. Pyruvate and oxygen are then transported into the mitochondria, where additional metabolic reactions occur. Pyruvate is converted to acetyl-CoA, enters the TCA cycle, and produces reducing equivalents, such as NADH and FADH_2_. These molecules feed into the electron transport chain, driving oxidative phosphorylation and ATP synthesis.

The subsequent sections describe the outcomes of these metabolic processes within each cellular compartment, along with the effects of varying glucose and oxygen influx patterns and the number of mitochondria. Furthermore, the initial molecular counts of each component were estimated based on the water molecule numbers reported in Supplementary Figures S2 and S3 (H_2_O:= = 1 × 10^13^ molecules in the cytoplasm; H_2_O:: = 1 × 10^11^ molecules in the mitochondria)^47^.

### From glucose transport to mitochondrial ATP synthesis

**Fig. 4:**
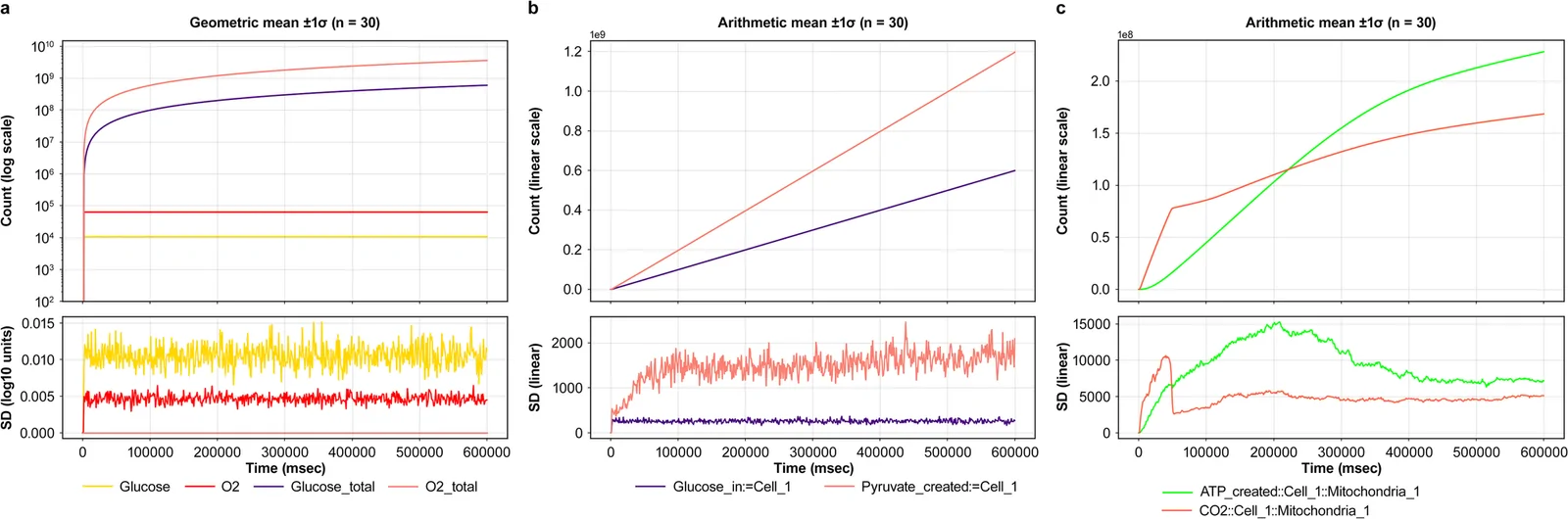
Each panel presents the mean and standard deviation calculated from *n* = 30 simulations. Although the standard deviations are also plotted in the upper panels, they are too small to be visually distinguishable. (**A**) Glucose, O_2_, total glucose influx (glucose_total), and total O_2_ influx (O2_total) in the extracellular environment. (**B**) Glucose influx (glucose_in:=Cell_1) and pyruvate production (Pyruvate_created:=Cell_1) in the cytoplasm, plotted on a linear scale. (C) ATP production (ATP_created::Cell_1::Mitochondria_1), and CO_2_ (CO_2_::Cell_1::Mitochondria_1) in the mitochondria, plotted on a linear scale.

Figure 4A presents the computational results obtained using the input files from Supplementary Figures S1–S3. The figure shows glucose and O_2_ concentrations in the extracellular environment up to 600 s, together with cumulative glucose influx (glucose_total) and oxygen influx (O2_total). As described earlier, glucose and oxygen continuously enter the system; glucose is transported into the cell by glucose transporters (glucose_transport:=), while oxygen enters by diffusion. Both gradually accumulate in the intracellular space, with a fraction remaining in the extracellular environment.

Figure 4B compares intracellular pyruvate production with glucose uptake, showing that nearly twice as much pyruvate is generated relative to glucose influx. Figure 4C illustrates the time course of cumulative ATP synthesis by ATP synthase, driven by proton translocation into the mitochondrial intermembrane space via the electron transport chain, alongside cumulative CO_2_ production from the TCA cycle. Metabolite yields normalized per unit of glucose uptake are shown in Fig. 3. These results show that 200 units of pyruvate were produced in the cytoplasm per 100 unit of glucose, of which 10.63 unit entered the mitochondria. Moreover, 85.94 units of protons were translocated into the mitochondrial intermembrane space, resulting in the mitochondrial production of 14.86 units of ATP. In addition, from the results in Fig. 4, the ATP production rate by ATP synthase calculated over the interval from 500 to 600 s was 155 molecules s^− 1^ per ATP synthase. This value is consistent with experimentally reported turnover rates of ATP synthase, which are typically on the order of 10^2^ molecules s^− 1^ per enzyme under physiological conditions^43^. The dynamics of additional key metabolites are shown in Supplementary Figures S4 and S5.

In the NNS framework, rate constants represent normalized probabilities of a reaction occurrence during a simulation time step, rather than classical kinetic parameters used in differential equation models. Consequently, the obtained ATP production rate depends not only on the rate constants assigned to individual reactions but also on the normalization factors and the simulation time scale. Therefore, the resulting reaction rates are inherently dependent on the chosen time unit and the molecular number scaling applied in the simulation.　In this study, the rate constants were assigned heuristically to yield physiologically plausible fluxes within the simplified reaction network. In particular, the parameters were adjusted such that the resulting ATP production rate fell within the experimentally reported range of several hundred molecules s^− 1^ per ATP synthase.

### Extension of the simulation time and sensitivity analysis

Supplementary Figure S6–S8 presents the results of extending the simulation for an additional 6e5 time units, beginning from the state at t = 6e5 described in the section entitled “From glucose transport to mitochondrial ATP synthesis.” Figures S6A–S6C show that the quantities exhibit trends similar to those shown in Fig. 4. Supplementary Figure S9 presents a portion of the corresponding input file. Since the files were already integrated, they were combined into a single file, where the initial values of the elements were set to those obtained at t = 6 × 10^5^ in the previous simulation. Consequently, only the simulation time parameter required adjustment for recalculation.

**Fig. 5:**
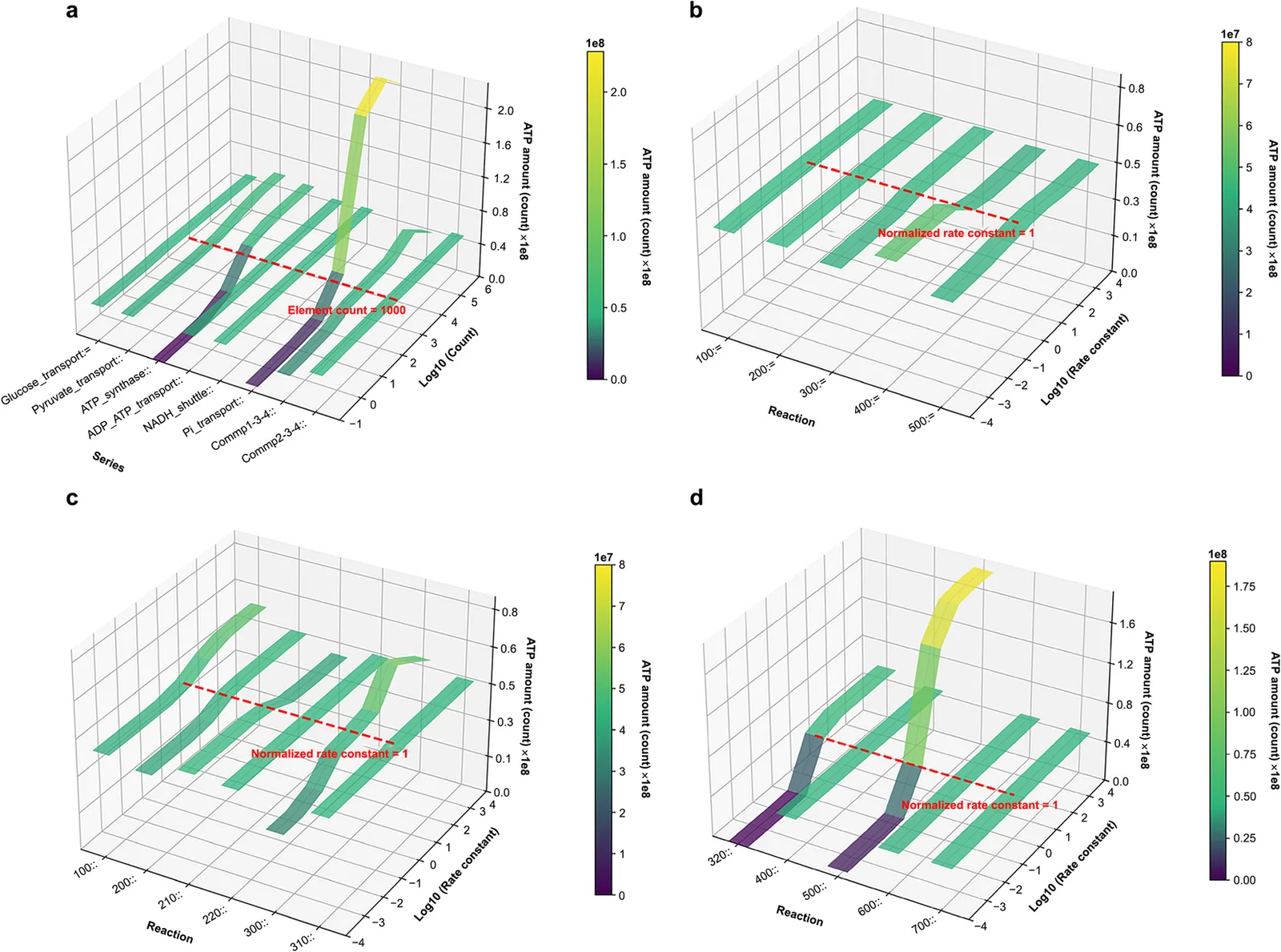
Results of the parameter study. Simulations were conducted using restart files generated at 600 s, and in each simulation only one parameter was varied at a time to evaluate differences in mitochondrial ATP production. The vertical axis in each panel represents the amount of ATP produced between 600 and 900 s. The rate constants shown are normalized values based on the reference set used in this study. (**A**) Effect of varying the number of each membrane protein from 0 to 1,000,000. (**B**) Effect of changing the rate constants of reactions 100:=−500:= by four orders of magnitude up and down. (**C**) Effect of changing the rate constants of reactions 100::−310:: by four orders of magnitude up and down. (**D**) Effect of changing the rate constants of reactions 320::−700:: by four orders of magnitude up and down.

A sensitivity analysis was performed using the restart file from Supplementary Fig. 9. By modifying one parameter at a time—membrane-protein copy numbers (0–1,000,000) or reaction rate constants (± 4 orders of magnitude)—we evaluated changes in ATP production during a 300-s simulation (Fig. 5). ATP synthase abundance and the rate constants of ATP synthesis (320::) and phosphate–proton symport (500::) exhibited the strongest influence on ATP output.

### Effects of different modes of glucose addition on ATP production

In the preceding simulations, glucose entered the system at a constant rate of 1 × 10^3^ molecules/ms. Subsequently, we investigated a scenario in which the total supplied glucose remained constant (6 × 10^8^ molecules) but was delivered in two separate pulses. This calculation was performed by modifying part of the restart file described in the section entitled “Extension of the simulation time and sensitivity analysis.”

**Fig. 6:**
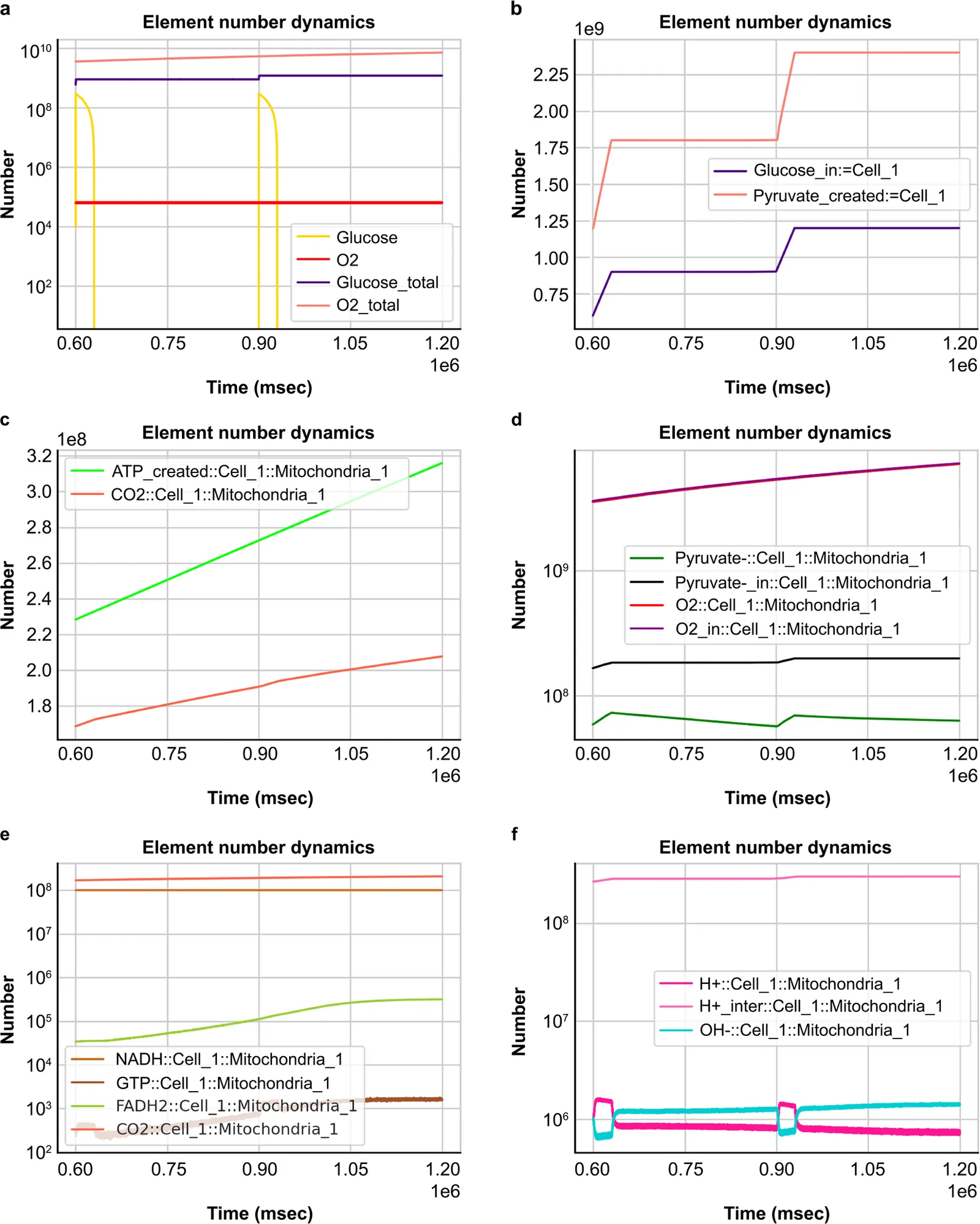
Results from a simulation in which glucose was supplied to the extracellular environment in two discrete pulses (3 × 10^8^ molecules each). (**A**) Glucose (glucose), O_2_ (O2), total glucose influx (glucose_total), and total O_2_ influx (O2_total) in the extracellular environment. (**B**) Glucose influx (glucose_in::=Cell_1) and pyruvate production (Pyruvate_created::=Cell_1) in the cytoplasm, plotted on a linear scale. (**C**) ATP production (ATP_created::Cell_1::Mitochondria_1) and CO_2_ (CO2::Cell_1::Mitochondria_1) in the mitochondria. (**D**) Pyruvate (Pyruvate−:=Cell_1::Mitochondria_1), pyruvate influx (Pyruvate_in:=Cell_1::Mitochondria_1), O_2_ (O2::Cell_1::Mitochondria_1), and O_2_ influx (O2_in::Cell_1::Mitochondria_1) in the mitochondria. (**E**) NADH (NADH::Cell_1::Mitochondria_1), GTP (GTP::Cell_1::Mitochondria_1), FADH_2_ (FADH2::Cell_1::Mitochondria_1), and CO_2_ (CO2::Cell_1::Mitochondria_1). (**F**) H+ (H+::Cell_1::Mitochondria_1), H+ in the intermembrane space (H+_inter::Cell_1::Mitochondria_1), and OH− (OH−::Cell_1::Mitochondria_1) in the mitochondria.

Specifically, 6 × 10^8^ molecules were divided into two pulses: 3 × 10^8^ molecules at the start of the simulation and the remaining 3 × 10^8^ molecules at t = 9 × 10^5^. The results are presented in Fig. 6, while a portion of the corresponding input file is shown in Supplementary Figure S10. Figure 6A shows that extracellular glucose levels decrease linearly over time. Figures 6B **and C** show that pyruvate production (Pyruvate_created::=Cell_1) and mitochondrial ATP production (ATP_created::Cell_1::Mitochondria_1). Figures 6D–F also show changes in other substrates. In this case, some substrates, such as pyruvate, respond immediately to the addition of glucose, whereas mitochondrial ATP production is only minimally affected.

### Effect of mitochondrial number on ATP production

**Fig. 7:**
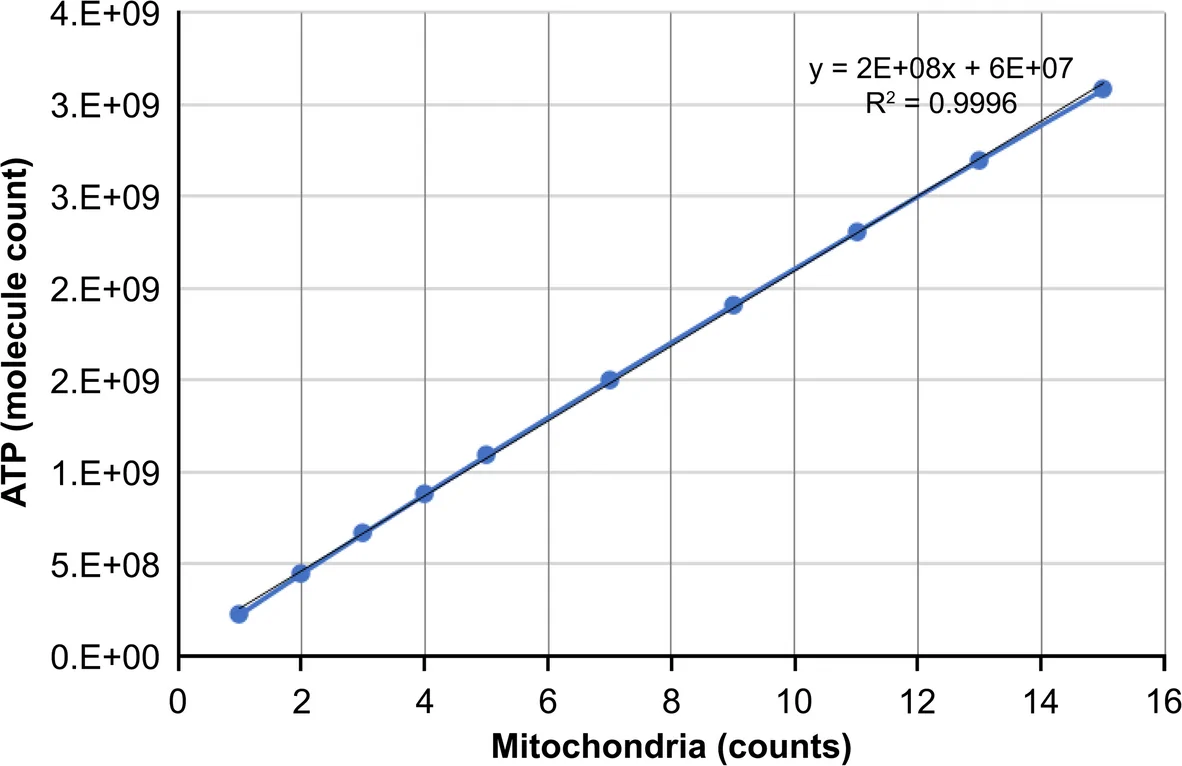
**C**hanges in total ATP production as a function of the number of mitochondria.

The NNS framework enables variations in both the number of cells and mitochondria. In the study, only the number of mitochondria was altered. Figure 7 shows the total mitochondria ATP production during the simulation period, from 0 to 6 × 10^5^ ms, as a function of mitochondrial count. Supplementary Figure S11 provides the system file for the control simulation, in which fifteenth mitochondria were assigned to a single cell. In the present system, the cytoplasm contains ample pyruvate and oxygen; therefore, even when the number of mitochondria increases, substrate supply remains sufficient, and the total cellular ATP production increases almost linearly.

### Effects of deuterium on ATP synthase activity

The potential influence of naturally occurring deuterium on ATP production has been previously investigated in several studies^48–50^. In this study, the model described in the section entitled “From glucose transport to mitochondrial ATP synthesis” was extended to evaluate possible effects of deuterium on the mitochondrial electron transport chain and ATP synthesis.

**Fig. 8:**
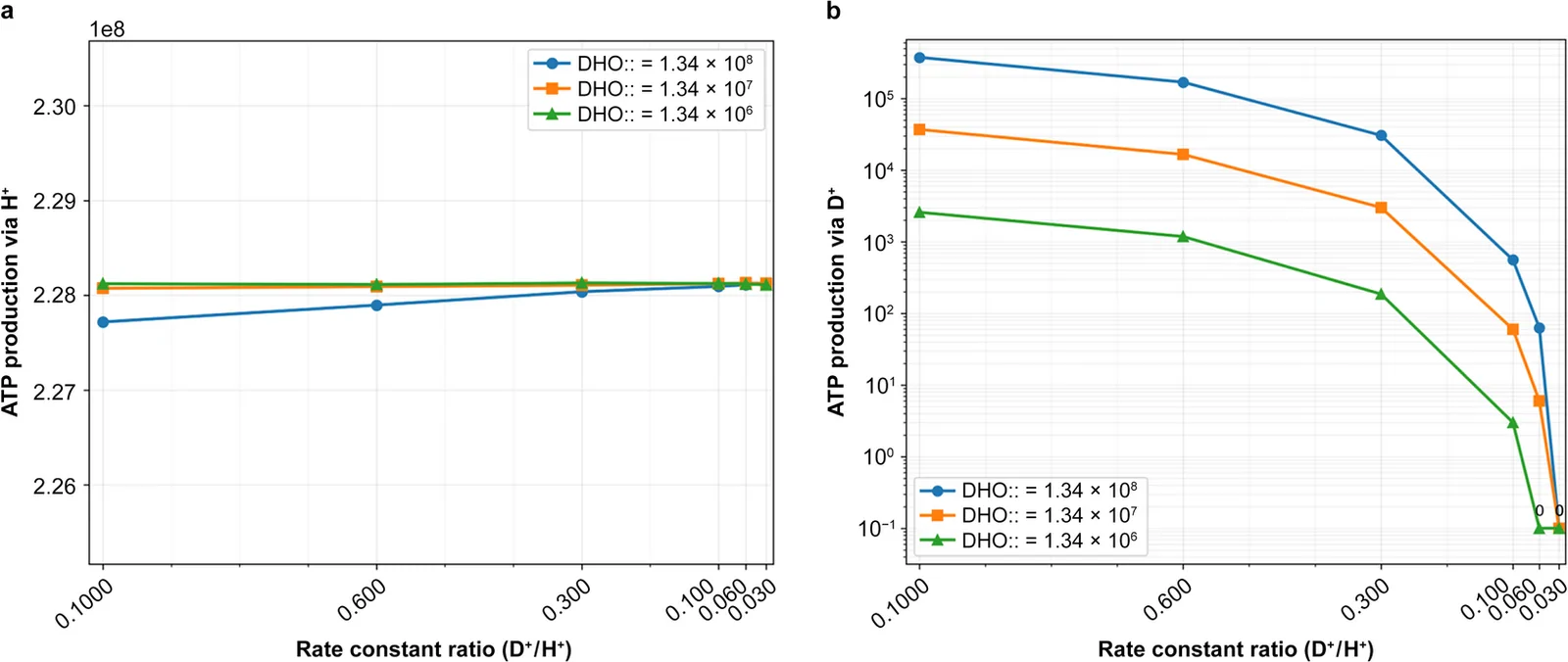
Effect of changes in the rate constants of D^+^-related reactions in ATP synthase. ATP production was evaluated when the rate constants of reactions involving D⁺ were reduced relative to those of the corresponding H^+^ reactions. Three reactions were considered: two electron transport reactions (rM_300:: and rM_310::) and the ATP synthesis reaction (rM_320::). For the deuterium case, corresponding reactions with reduced rate constants (rM_301::, rM_311::, and rM_321::) were introduced. The horizontal axis shows the rate constant ratio (D^+^ / H^+^). The numbers of DHO molecules used in the simulations were estimated based on the D^+^/H^+^ ratio (~ 1/15,000) reported in biological systems^49^. (**A**) ATP production driven by H^+^.(**B**) ATP production driven by D^+^. The number of DHO molecules was varied in the simulations (3.12 × 10^8^, 3.12 × 10^7^, and 3.12 × 10^6^) to examine the influence of deuterium abundance. For rate constant ratios of 0.06 and 0.03, values equal to zero are plotted as 0.1 for visualization purpose.

To represent deuterium participation, D^+^-dependent reactions corresponding to the two electron transport reactions and the ATP synthesis reaction were introduced alongside the original H^+^-dependent reactions. In these reactions, the rate constants were uniformly reduced relative to those of the corresponding H^+^ reactions to examine the effect of slower proton transfer by deuterium. The details of the extended model are presented in Supplementary Figure S12 and Supplementary Table S1. Table 3 summarizes the differences between the H^+^- and D^+^-driven ATP production models, and the corresponding simulation results are shown in Fig. 8.

**Table 3:** Comparison of parameters and reaction characteristics between the H^+^- and D^+^-driven ATP production models.

Feature	H^+^ model	D^+^ model
Proton species	H+	D⁺
Water species	H_2_O	DHO
ETC reactions	rM_300::, rM_310::	rM_301::, rM_311::
ATP synthase reaction	rM_320::	rM_321::
Rate constant	k_H	Reduced relative to k_H
Rate constant ratio	1.0	1.0–0.03
Biological interpretation	Normal proton transfer	Slower deuteron transfer
ATP production	Dominant	Substantially lower
Intermembrane ion abundance	H+_inter dominant	D^+^_inter substantially lower

The amount of deuterated water in mitochondria was estimated based on the physiological abundance ratio of deuterium (D^+^/ H^+^ ≈ 1/15000)^49^. Accordingly, the number of DHO molecules was varied around 1.34 × 10^7^, using values of 1.34 × 10^8^, 1.34 × 10^7^, and 1.34 × 10^6^ in the simulations.

As shown in Fig. 8A, ATP production driven by H^+^ was largely unaffected by the presence of deuterium and variations in the rate constants of deuterium-associated reactions. In contrast, ATP production driven by D^+^ exhibited strong dependence on deuterium numbers and the corresponding reaction rate constants, and remained substantially lower than that driven by protons.

## Discussion

The present model consistently and quantitatively simulated the pathway from glucose uptake to ATP production through oxidative phosphorylation.

The NNS framework is related to stochastic simulation approaches used in biochemical modeling, such as the Gillespie stochastic simulation algorithm and stochastic Petri nets. However, NNS directly evaluates reaction events using binomial probabilities within hierarchical domain structures, thereby enabling the implementation of complex compartmental models without the need to explicitly derive stochastic rate equations.

In constructing a metabolic model, imbalances in cofactors must be critically avoided. While the carbon, oxygen, and hydrogen atoms of glucose are metabolized and converted into water and carbon dioxide, intracellular cofactors, such as ATP and NAD^+^, must undergo cyclical interconversion. Specifically, ATP and NAD^+^ are transformed into ADP and inorganic phosphate and NADH and are subsequently regenerated into their original forms. In the current model, ATP synthesized in the mitochondria was transported to the cytoplasm via the mitochondrial ADP/ATP translocase (600::) in Table 2, where it was consumed in cytoplasmic via ATP hydrolysis (500:=) in Table 1 and reconverted to ADP and inorganic phosphate. ATP synthesis in mitochondria—as represented by reaction (320::) in Table II—is driven by proton translocation through ATP synthase, a rotary molecular machine that converts electrochemical energy into chemical energy^51^. Similarly, NADH and FADH_2_ produced in the TCA cycle were oxidized in the electron transport chain, thereby generating NAD^+^ and FAD. To sustain glycolysis in the cytoplasm, via the lactate dehydrogenase (LDH) reaction (400:=) in Table 1 consumed NADH to produce NAD^+^. Simultaneously, a mitochondrial shuttle mechanism enabled the transport of NAD^+^ into the cytoplasm and the return of NADH to the mitochondria^52^. This stoichiometric representation within the NNS framework offers an intuitive understanding of the interconnectedness of individual reactions.

Although the NNS framework primarily handles element numbers, it can also incorporate compartmental volumes. In the current model, the number of water molecules was defined for both the cytoplasm and mitochondria, reflecting the contribution of water of approximately 70% to cellular mass. Using these values as reference points, cell and mitochondrial sizes were estimated to be 1 × 10^13^ water molecules in the cytoplasm and 1 × 10^11^ in the mitochondria. Based on these calculations, the approximate abundances of other substrates could also be inferred^53^. Furthermore, employing water molecule numbers as normalization constants within the algorithm eliminated the variability introduced by the numerical scale of rate constants (see *Reaction entries in Supplementary Figures S2 and S3B).

Because the NNS framework is specifically designed for dynamic analysis, reproducing a strict steady state, defined as a condition in which the abundance of a given element remains constant or increases at a uniform rate, requires the precise determination of rate constants via optimization. In the present simulations, several elements, such as pyruvate (Supplementary Figures S7A, and S8A), NAD^+^ (Supplementary Figures S7B and S8C), and CoA (Supplementary Figure S8F), had not yet reached a steady state after the restart calculation. Nonetheless, the mitochondrial production rates of pyruvate and ATP remained nearly constant (Supplementary Fig. 6C), demonstrating biological homeostasis in which steady production is maintained despite fluctuations in internal states.

Deuterium was incorporated into the model, as discussed in the section entitled “Effects of deuterium on ATP synthase activity.” The natural abundance ratio of deuterium to hydrogen in biological systems is approximately D^+^/ H^+^ ≈ 1/15,000, and the estimated probability of deuteron participation in protonation processes is on the order of 10^− 4^–10^− 5^^49^. The simulations indicated that ATP production driven by D^+^ is strongly influenced by the number of deuterium atoms and the associated reaction rate constants, whereas that driven by H^+^ remains largely unaffected by deuterium.

Extensive simultaneous deuteration of multiple c subunits was considered unlikely in the present model as the calculated D^+^_inter concentration remained substantially lower than H^+^_inter under all simulated conditions. In the calculations shown in Fig. 8, the D^+^_inter/H^+^_inter ratio in the mitochondrial intermembrane space was approximately 8 × 10^− 4^, even under the highest deuterium condition examined (DHO = 1.34 × 10^8^ and rate constant ratio = 1). Therefore, explicit modeling of F_0_ subunit turnover or half-life was not included in this study.

Considering the role of deuterium in biological reactions is important for understanding the metabolic mechanisms, as isotopic fractionation of hydrogen has been reported to vary substantially across different metabolic pathways^50^. In the present study, the analysis was limited to three reactions associated with the electron transport chain and ATP synthesis. However, the effects of deuterium in substrates and cofactors, as well as potential differences in transport efficiency mediated by various membrane proteins, could also be examined in future studies.

The flexibility and extensibility of the NNS framework are expected to facilitate further exploration of such complex metabolic systems. Further refinement will be required to fully capture the complexity of intracellular reactions, including the incorporation of ion buffering and more detailed reaction formulations. Nevertheless, the flexibility and scalability of the NNS framework demonstrates its potential as a tool for analyzing biochemical systems.

## Supplementary Information

Below is the link to the electronic supplementary material.

Supplementary Material 1

## Data Availability

The input files and scripts used in this study are available on GitHub at https:/github.com/taka-b/binomial/tree/e47cbdc (branch: binomial_v018_02). Because the simulation output files are large, they are available from the corresponding author upon reasonable request.

## Abbreviations

H_2_O: Water
H^+^: Proton
OH^−^: Hydroxide ion
DHO: Deuterated water species
D⁺: Deuteron
OD⁻: Deuteroxide ion
CoA-SH: Coenzyme A (reduced form)
Acetyl_CoA: Acetyl coenzyme A
NAD^+^: Nicotinamide adenine dinucleotide (oxidized form)
NADH: Nicotinamide adenine dinucleotide (reduced form)
FAD: Flavin adenine dinucleotide (oxidized form)
FADH_2_: Flavin adenine dinucleotide (reduced form)
GDP: Guanosine diphosphate
GTP: Guanosine triphosphate
ADP: Adenosine diphosphate
ATP: Adenosine triphosphate
Pi: Inorganic phosphate
H^+^_Inter: Proton in the intermembrane space
NADH_shuttle: NADH shuttle
Pi_transport: Phosphate transporter
ADP_ATP_transport: Adenine nucleotide translocator
ATP_synthase: ATP synthase complex
ATP: Adenosine triphosphate
Commp1-3-4: Respiratory chain supercomplex (Complexes I, III, and IV)
Commp2-3-4: Respiratory chain supercomplex (Complexes II, III, and IV)
O_2_: Molecular oxygen
CO_2_: Carbon dioxide
