# Supplementary material for: Numerical simulation of mitochondrial systems for ATP generation and membrane transport
Source: Sci Rep. 2026 Jul 20;16:33003. doi: 10.1038/s41598-026-61463-0 (PMC13680276; doi:10.1038/s41598-026-61463-0)
Supplement: Supplementary file 1 [file 41598_2026_61463_MOESM1_ESM.docx]

**Numerical Simulation of Mitochondrial Systems for ATP Generation and Membrane Transport**

Takashi Sato*^1)^, Tsuyoshi Osawa^2) 3) 4)^

*1) Research & Development Center, Zeon Corporation, Kanagawa, 210-9507 Japan, email: sato@zeon.co.jp

2) Research Center for Advanced Science and Technology, The University of Tokyo, Tokyo, 153-8904

3) Department of Chemistry and Biotechnology, Graduate School of Engineering, The University of Tokyo, Tokyo 113-0033, Japan

4) Department of Biological Sciences, Graduate School of Science, The University of Tokyo, Tokyo, 113-0033, Japan

**Supplementary Methods**

**Random number generation**

In the NNS simulations, stochastic reaction events were determined using binomial sampling implemented in NumPy (numpy.random.binomial). In addition, Gaussian-distributed random numbers generated using Python’s random.gauss function were used in certain approximations of stochastic fluctuations. Pseudo-random numbers were generated using the default random number generators of NumPy and the Python standard library. Because a fixed random seed was not imposed, independent runs produce slightly different trajectories while preserving the overall statistical behavior.

**Supplementary Figures**

**Supplementary Figure S1.** Input file used for the simulation, in which glucose (glucose_in) was supplied to the environment at each calculation step (1 × 10^3^ molecules each), and O_2_ (O2_total) was supplied at each calculation step (6 × 10^3^ molecules). This file enables the visualization of the total amounts of glucose and O₂ entering the system. The variables *glucose_total* and *O2_total* denote the cumulative numbers of molecules introduced into the cellular environment.

**Supplementary Figure S2**. Cell definition file corresponding to the system file shown in Figure 3 at the Set_01 statement. The Element statement specifies the main element names and their initial quantities as defined in the file. The “:=” symbol indicates the location within the cell. The initial values listed next to each name represent only molecular counts. The Reaction statement defines reactions within the cell, including reaction names, reactants with their orders, products with their order, and associated rate constants. The rate constants are dimensionless and are provided as numerical values only.

**Supplementary Figure S3**. The Set-02 file defining the mitochondria within the system file.

(A) The element names and their initial quantities are specified by the Set_02 statement. The “::” symbol denotes the location within the mitochondria. The initial values indicate molecular counts only.
(B) Reactions occurring in the mitochondria are listed, including reaction names, reactants and their stoichiometric order, products and their stoichiometric orders, and rate constants. The rate constants are dimensionless and expressed solely as numerical values.

**Supplementary Figure S4**. Additional results derived from the input files shown in Supplementary Figures S1-S3. All results represent the mean values obtained from *n* = 30 simulations, with standard deviations corresponding to ±3σ. The ±3σ ranges are indicated, and they are visually discernible for OH^-^ (OH-:=Cell_1) in panel (C), and (C) and for O₂ (O2:=Cell_1) in panel (C). The stochastic fluctuations arise from binomial sampling of reaction events in the NNS framework (see Supplementary Methods).
(A) Glucose (glucose:=Cell_1), pyruvate (Pyruvate-:=Cell_1), and lactate (Lactic-:=Cell_1) counts in the cytoplasm.
(B) NADH (NADH:=Cell_1) and NAD⁺ (NAD+:=Cell_1) levels in the cytoplasm, plotted on a linear scale.

(C) H^+^ (H+:=Cell_1), OH^-^ (OH-:=Cell_1), and O₂ (O2:=Cell_1) counts in the cytoplasm.
(D) ATP (ATP:=Cell_1), ADP (ADP:=Cell_1), and Pi (Pi:=Cell_1) levels in the cytoplasm.

**Supplementary Figure S5.** Results generated from the input file presented in Supplementary Figures S1–S3. All plotted elements are located within the mitochondria. All results represent the mean values obtained from *n* = 30 simulations, with standard deviations corresponding to ±3σ.
The ±3σ ranges are visually discernible for OH⁻ (OH– ::Cell_1::Mitochondria_1) in panel (B), NAD⁺ (NAD+::Cell_1::Mitochondria_1) in panel (C), and GDP (GDP ::Cell_1::Mitochondria_1) in panel (C). The stochastic fluctuations arise from binomial sampling of reaction events in the NNS framework (see Supplementary Methods).

(A) Pyruvate (Pyruvate-:=Cell_1 ::Mitochondria_1), pyruvate influx (Pyruvate_in:=Cell_1 ::Mitochondria_1), and O₂ (O2::Cell_1::Mitochondria_1).
(B) H⁺ (H+::Cell_1::Mitochondria_1), H⁺ in the intermembrane space (H+_inter::Cell_1::Mitochondria_1), and OH⁻ (OH– ::Cell_1::Mitochondria_1).
(C) NAD⁺ (NAD+::Cell_1::Mitochondria_1), GDP (GDP::Cell_1::Mitochondria_1), and FAD (FAD::Cell_1::Mitochondria_1).

(D) NADH (NADH::Cell_1::Mitochondria_1), GTP (GTP::Cell_1::Mitochondria_1), and FADH₂ (FADH2::Cell_1::Mitochondria_1)
(E) ADP (ADP::Cell_1::Mitochondria_1 ), ADP (ADP::Cell_1::Mitochondria_1), and Pi (Pi:=Cell_1 ::Mitochondria_1).

(F) CoA (CoA-SH::Cell_1::Mitochondria_1), and acetyl-CoA (Acetyl_CoA::Cell_1::Mitochondria_1), and CO₂ (CO2::Cell_1::Mitochondria_1).

**Supplementary Figure S6**. Results obtained using a restart file generated from the data at 600 s in Figure 4A. The restart file was created by combining the corresponding three input files used for Figure 4A.

(A) Glucose (glucose), O₂ (O2), total glucose influx (glucose_total), and total O₂ influx (O2_total) in the extracellular environment. (B) Glucose influx (glucose_in:=Cell_1) and pyruvate production (Pyruvate_created:=Cell_1) in the cytoplasm, plotted on a linear scale. (C) ATP production (ATP_created::Cell_1::Mitochondria_1), and CO₂ (CO2::Cell_1::Mitochondria_1) in the mitochondria, plotted on a linear scale.

**Supplementary Figure S7**. Results obtained using the same restart file as in Supplementary Figure 6. All variables shown in panels (A)–(D) represent cytoplasmic counts.

(A) O₂ (O2:=Cell_1), glucose (glucose:=Cell_1), pyruvate (Pyruvate-:=Cell_1), and lactate (Lactic-:=Cell_1).
(B) NADH (NADH:=Cell_1) and NAD⁺ (NAD+:=Cell_1), plotted on a linear scale.

(C) OH^-^ (OH-:=Cell_1), and H^+^ (H+:=Cell_1), and H_2_O (H2O:=Cell_1).
(D) ATP (ATP:=Cell_1), ADP (ADP:=Cell_1), and Pi (Pi:=Cell_1).

**Supplementary Figure S8.** Results obtained using the same restart file as in Supplementary Figure 6. All elements plotted here are located in the mitochondria.
(A) Pyruvate (Pyruvate-:=Cell_1 ::Mitochondria_1), pyruvate influx (Pyruvate_in:=Cell_1 ::Mitochondria_1), O₂ (O2::Cell_1::Mitochondria_1), and O₂ influx (O2_in::Cell_1::Mitochondria_1).
(B) H⁺ (H+::Cell_1::Mitochondria_1), H⁺ in the intermembrane space (H+_inter::Cell_1::Mitochondria_1), and OH⁻ (OH– ::Cell_1::Mitochondria_1).
(C) NAD⁺ (NAD+::Cell_1::Mitochondria_1), GDP (GDP::Cell_1::Mitochondria_1), and FAD (FAD::Cell_1::Mitochondria_1).

(D) NADH (NADH::Cell_1::Mitochondria_1), GTP (GTP::Cell_1::Mitochondria_1), FADH₂ (FADH2::Cell_1::Mitochondria_1), and CO₂ (CO2::Cell_1::Mitochondria_1).
(E) ADP (ADP::Cell_1::Mitochondria_1 ), and Pi (Pi:=Cell_1 ::Mitochondria_1), and Pi influx (Pi_in:=Cell_1 ::Mitochondria_1).

(F) CoA (CoA-SH::Cell_1::Mitochondria_1), and acetyl-CoA (Acetyl_CoA::Cell_1::Mitochondria_1).

**Supplementary Figure S9.** Opening section of the restart file generated from the input file used for the calculations in Figure 4. The simulation period was extended from *t* = 6e5 to *t* = 12e5, while all other conditions remained unchanged. As this is a restart file, the initial values of each element correspond to the final values obtained in Figure 4. Definitions of the cell and mitochondria are omitted here because only the initial values differed, whereas all other parameters remained constant.

Variable names and notations are consistent with those in Supplementary Figure S1 (e.g., *glucose_total* and *O2_total* indicate cumulative influxes into the cellular environment).

**Supplementary Figure S10.** The first section of the practical restart file, where glucose was introduced into the environment in two discrete pulses (3 × 10^8^ molecules each). This file shows the total amounts of glucose and O₂ entering the system, with *glucose_total* and *O2_total* indicating the cumulative numbers of molecules entering the cellular environment.

**Supplementary Figure S11.** Input file used for the control simulation involving fifteen mitochondria.

**Supplementary Figure S12. Additional element and reaction definitions introduced to model the influence of deuterium in the mitochondrial system.**

This figure shows only the newly added elements and reactions used in the extended model that incorporates deuterium-related processes described in the section entitled “**Effects of deuterium on ATP synthase activity”**.

(A) Additional element definitions including deuterium-containing species. The element names and their initial quantities are specified by the *Element* statement. The “::” symbol denotes the mitochondrial location. Deuterium-containing species such as DHO (semi-heavy water, HDO), OD⁻, and D⁺ are included together with their corresponding intermediate states.

(B) Additional reaction definitions involving deuterium in the mitochondrial system. These reactions describe proton/deuteron exchange between water species and deuterium-dependent electron transport and ATP synthesis processes. Reaction entries list the reaction identifiers, reactants and their stoichiometric orders, products and their stoichiometric orders, and the associated rate constants.

**Supplementary Table**

**Supplementary Table 1.**

**Supplementary Table S1. Rate constants used for deuterium-related proton transfer reactions.**

Rate constants used for deuterium-associated proton transfer reactions in the simulations shown in Figure 8. The values were scaled according to the relative proton transfer efficiency ratio (D⁺/H⁺). Reactions (301::) and (311::) represent deuterium-dependent proton translocation associated with the electron transport chain, whereas reaction (321::) represents ATP synthesis driven by deuteron translocation through ATP synthase.

Reaction	Ratio = 1	0.6	0.3	0.1	0.06	0.03

rM_301::	1.0 × 10⁵	6.0 × 10⁴	3.0 × 10⁴	1.0 × 10⁴	6.0 × 10³	3.0 × 10³

rM_311::	1.0 × 10⁵	6.0 × 10⁴	3.0 × 10⁴	1.0 × 10⁴	6.0 × 10³	3.0 × 10³

rM_321::	6.0 × 10³	3.6 × 10³	1.8 × 10³	6.0 × 10²	3.6 × 10²	1.8 × 10²
